# A message from the New Editor-in-Chief

**DOI:** 10.2188/jea.18.1

**Published:** 2008-02-28

**Authors:** Tomotaka Sobue

**Affiliations:** Cancer Information Services and Surveillance Division, Center for Cancer Control and Information Services, National Cancer Center

I am honored to assume the responsibility of the Editor-in-Chief (EIC) of the Journal of Epidemiology. For the last 6 years, 2002-2007, the editorial committee has been successfully managed under the strong leadership of the former EIC, Dr. Yosikazu Nakamura. During his term as the EIC, the journal has achieved the fundamentals required to become an international scientific journal. The journal successfully obtained an impact factor in 2005 in the first attempt and scored 1.240 in 2006. The journal has been issued bimonthly since 2000 and distributes over 30 articles annually. Its successful management is reflected in the extraordinarily speedy responses after article submission: the first reviewers' comments reach authors within an average of 18 days since the receipt of an article.

The journal will continue to serve the research community across a broad range of exciting developments in epidemiologic research. First, I ask all members of the Japan Epidemiological Association to continually contribute to the journal by submitting significant research experiments. However, this alone is insufficient to further establish our excellence as an international scientific journal. Although based in Japan, the journal welcomes contributions from all over the world, especially from Asian countries. The importance and significance of the work it publishes is reflected in its international subscriptions and readership outside Japan. In order to maintain this high standard, the editorial committee will continue to make every effort to improve the quality of the journal.

My own field of study is cancer epidemiology. However, I can assure you that the journal is not about to become a journal of cancer. We shall publish the best of all epidemiologic researches.

Tomotaka Sobue, MD, MPH Editor-in-Chief The Journal of Epidemiology  Cancer Information Services and Surveillance Division Center for Cancer Control and Information Services National Cancer Center 5-1-1 Tsukiji, Chuo-ku, Tokyo 104-0045, Japan  Phone: +81-3-3542-2511, ext: 3424 Fax: +81-3-3546-0630 E-mail: edit-je@cied2.res.ncc.go.jp

